# Long-term Rotarix Vaccine Effectiveness in Coastal Kenya, Estimated From Child Hospital Admissions Data From July 2014 to December 2022

**DOI:** 10.1093/ofid/ofag674

**Published:** 2026-10-10

**Authors:** Zadock Kipkirui, Charity Wamwea, Esther N Katama, Robinson Cheruiyot, Martin Mutunga, Agustus Kea, Arnold W Lambisia, D James Nokes, Umesh D Parashar, Jacqueline E Tate, Eunice W Kagucia, Charles N Agoti

**Affiliations:** Kenya Medical Research Institute–Wellcome Trust Research Programme, Kilifi, Kenya; Department of Statistics and Actuarial Science, Jomo Kenyatta University of Agriculture and Technology, Nairobi, Kenya; Department of Statistics and Actuarial Science, Jomo Kenyatta University of Agriculture and Technology, Nairobi, Kenya; Kenya Medical Research Institute–Wellcome Trust Research Programme, Kilifi, Kenya; Kenya Medical Research Institute–Wellcome Trust Research Programme, Kilifi, Kenya; Kenya Medical Research Institute–Wellcome Trust Research Programme, Kilifi, Kenya; Kenya Medical Research Institute–Wellcome Trust Research Programme, Kilifi, Kenya; Kenya Medical Research Institute–Wellcome Trust Research Programme, Kilifi, Kenya; Kenya Medical Research Institute–Wellcome Trust Research Programme, Kilifi, Kenya; Viral Gastroenteritis Branch, Division of Viral Diseases, National Center for Immunization and Respiratory Diseases, Centers for Disease Control and Prevention, Atlanta, Georgia, USA; Viral Gastroenteritis Branch, Division of Viral Diseases, National Center for Immunization and Respiratory Diseases, Centers for Disease Control and Prevention, Atlanta, Georgia, USA; Kenya Medical Research Institute–Wellcome Trust Research Programme, Kilifi, Kenya; Kenya Medical Research Institute–Wellcome Trust Research Programme, Kilifi, Kenya; Department of Public Health, Pwani University, Kilifi, Kenya

**Keywords:** effectiveness, Kenya, Rotarix, rotavirus, test-negative

## Abstract

**Background:**

Two doses of Rotarix demonstrated approximately 64% effectiveness against rotavirus hospitalization in Kenyan children 2.5 years postintroduction. We evaluated long-term Rotarix effectiveness and monitored circulating genotypes over its 8.5 years of use in Kenya.

**Methods:**

We conducted a test-negative case-control study in coastal Kenya among children admitted to Kilifi County Referral Hospital with diarrhea from July 2014 to December 2022. Eligible children had known Rotarix vaccination status, parent/guardian consent, and rotavirus testing via enzyme-linked immunosorbent assay. Positives were genotyped by sequencing. Rotarix effectiveness was estimated using unconditional logistic regression, adjusting for age, sex, calendar year, seasonality, and nutritional status.

**Results:**

Of the 1104 children (median age, 12 months [interquartile range, 8–18 months]) included in the effectiveness analysis, 87% (962/1104) had received 2 doses, 6% (70/1104) 1 dose, and 7% (72/1104) zero doses. Rotavirus was detected in stool specimens of 147 (13%) children. Overall, adjusted 2-dose Rotarix effectiveness was 68% (95% confidence interval [CI], 42%–82%). Effectiveness in children aged <12 months was 83% (95% CI, 62%–93%) compared to 38% (95% CI, −106% to 78%) in children aged ≥12 months. Genotyping of 147 positive samples revealed G3P[8] (66/147 [44.9%]), G1P[8] (32/147 [21.8%]), and G2P[4] (23/147 [15.6%]) to be the most common circulating rotavirus genotypes during the Rotarix vaccination era.

**Conclusions:**

Rotarix maintained modest effectiveness in coastal Kenya during its 8.5 years of use. Effectiveness declined in the second year of life onward, probably reflecting a combination of both waning immunity and accumulation of natural immunity among unvaccinated children. Emergence of vaccine heterologous strains emphasizes the need for continued effectiveness surveillance.

## Teaser

Rotarix vaccine had 68% (95% CI, 42%–82%) effectiveness over 8.5 years in coastal Kenya. Effectiveness was high in infancy but declined in the second year and beyond, with partially/fully vaccine heterologous genotypes emerging, suggesting ongoing vaccine-driven genotype replacement.

In 2009, the World Health Organization (WHO) recommended inclusion of approved rotavirus vaccines in all national immunization programs (NIPs) [1]. Initially, 2 live-attenuated oral rotavirus vaccines were WHO prequalified: monovalent (G1P[8]) Rotarix (GlaxoSmithKline, Belgium) and pentavalent (G1, G2, G3, G4, and P1A[8]) RotaTeq (Merck & Co, USA). In 2018, a further 2 oral rotavirus vaccines were WHO prequalified: monovalent (G9P[11]) Rotavac (Bharat, India) and pentavalent (G1, G2, G3, G4, and G9) ROTASIIL (Serum Institute of India). As of June 2026, 142 countries had introduced 1 of the 4 WHO-prequalified rotavirus vaccines into their NIP [2]. However, ongoing diarrheal disease surveillance globally indicates that rotavirus is still a leading cause of diarrhea morbidity and mortality in children aged <5 years [3–5].

Kenya is a lower-middle-income country in East Africa with a population size of approximately 50 million people. With support from Gavi, the Vaccine Alliance, Kenya introduced the Rotarix vaccine into its NIP in July 2014 [4], which continued until December 2022, when the country switched to Rotavac vaccine following Rotarix supply disruption [6–8]. Before Rotarix introduction in Kenya, the country was among the top 10 sub-Saharan African countries that collectively accounted for approximately 65% of all rotavirus deaths globally [9, 10]. Local data from 2012 indicated that rotavirus disease had an annual incidence of approximately 3015 outpatient visits, approximately 279 hospitalizations, and approximately 65 deaths per 100 000 children aged <5 years [11].

During its use in Kenya, Rotarix was administered in 2 doses at 6 and 10 weeks of age. At 2.5 years postintroduction, a multisite study estimated its 2-dose vaccine effectiveness (VE) at approximately 64% (95% confidence interval [CI], 35%–80%), with similar point estimate of effectiveness in children aged ≥12 months (72% [95% CI, 10%–91%]) compared with those <12 months (67% [95% CI, 30%–84%]) [4]. In 2 demographic surveillance sites in Kenya (Kilifi and Siaya), an approximately 60% decline in rotavirus-associated hospitalizations and a >40% decline in all cause-diarrhea admissions were reported [12]. Whereas the G1P[8] strain was the predominant strain in Kenya pre–vaccine introduction, its prevalence appeared to diminish post–vaccine introduction while that of G2P[4] strains was increasing [4, 13].

Previous studies found lower effectiveness of oral rotavirus vaccines in high-mortality settings compared to low-mortality settings [14–17]. Furthermore, vaccine efficacy estimates obtained from clinical trials in several low- and middle-income countries [12–14] showed a 30%–40% drop in the level of protection from the first to the second year of life. Continued monitoring of effectiveness and the impact of these vaccines beyond the initial introduction years is critical to justify their continued use. This study utilizes long-term inpatient surveillance data from Kilifi County Referral Hospital (KCRH), coastal Kenya, to (1) evaluate the long-term effectiveness of Rotarix vaccine over its 8.5 years of use in the Kenyan NIP, and (2) investigate rotavirus strain distribution and potential genotype replacement before and during the Rotarix vaccination era.

## MATERIALS AND METHODS

### Study Design and Setting

We carried out a test-negative case-control analysis nested within the long-term pediatric acute gastroenteritis (AGE) surveillance study in KCRH [18, 19]. KCRH serves the rural/periurban population mostly residing in Kilifi County, coastal Kenya. The KCRH AGE surveillance study is run from the hospital pediatric ward, which has an approximately 40-bed capacity. Rotavirus surveillance in KCRH pediatric wards began in September 2009, has continued to date (July 2026), and is coordinated by the Kenya Medical Research Institute–Wellcome Trust Research Programme (KWTRP).

### Participants and Case Definition

Children aged <13 years whose parent/guardian reported diarrhea (defined as history of passing ≥3 loose watery stools within the previous 24 hours) were eligible for enrollment. Rotavirus vaccination status was ascertained through the Kilifi integrated Vaccine Monitoring Study [20], an electronic registry that captures childhood immunization in real time across clinics within the Kilifi Health and Demographic Surveillance System (KHDSS) area run by KWTRP [19]. Epidemiologic data including patient demographics (age, sex, location), clinical characteristics (diarrhea, vomiting, dehydration severity), vaccination status (Rotarix doses), and temporal factors (admission/discharge date) were collected from enrolled patients by a trained field staff and attending clinician and recorded into an electronic questionnaire.

### Laboratory Testing and Genotyping

Enrolled participants who had provided a stool sample for microbiological investigation on the request of attending clinicians were tested for rotavirus infection. Stool samples were transferred the same day to the KWTRP laboratory located adjacent to the hospital and stored at −80°C until their time for testing (frequently within a month). Testing was performed using enzyme-linked immunosorbent assay (ELISA; ProSPect Rotavirus Kit, Oxoid, Basingstoke, UK) that detects rotavirus group A (RVA) antigens. Rotavirus positive samples were subsequently genotyped (G [VP7] and P [VP4] typing) using partial or whole genome sequencing as previously described [21, 22].

### Sample Size

We retrospectively calculated that a sample size of 107 test-positive and 213 rotavirus test-negative participants (a case-to-control ratio of 1:2) was needed to have a power of 80% at 5% significance level to demonstrate a VE of ≥50% vaccine coverage of 70%. Further required sample size estimates at different vaccine coverage scenarios are provided in Supplementary Table 1.

### Statistical Analysis

We evaluated Rotarix (VE) using a test-negative case-control study design [23]. Eligibility criteria for inclusion in the current effectiveness analysis were as follows: (1) enrollment between July 2014 and December 2022; (2) age appropriate to have received Rotarix during this period (children born on or after 14 May 2014); (3) written informed consent provided by parent/guardian for the child to be included in research studies; (4) availability of their rotavirus test result; and (5) a completed metadata questionnaire (ie, child's age, vaccination status, other symptoms, and demographic information). Complete Rotarix vaccination was defined as having received 2 doses while partial Rotarix vaccination was defined as having received 1 dose.

Participants whose stool specimen tested rotavirus positive by ELISA were assigned case status while those whose specimens tested rotavirus negative were assigned control status. Children with unknown rotavirus status were excluded, as were those who were missing vaccination information. Baseline characteristics of cases and controls were compared using χ^2^ test (categorical variables) and Wilcoxon rank-sum test (continuous variables). We calculated odd ratios for vaccination among cases versus controls using unconditional logistic regression. Rotavirus vaccination was our main exposure variable of interest with Rotarix vaccination status defined as 2 versus 0 doses, at least 1 dose versus 0 doses, and exactly 1 dose versus 0 doses. The zero dose was our reference category when we fitted our model. The functional form of the fitted model is summarized below:


$$\log\;\left(\frac{\mathrm{Pr}(Y_{i}=1)}{\mathrm{Pr}(Y_{i}=0)}\right)=\beta_{0}+\beta_{1}\;x_{1i}+\beta_{2}\;x_{2i}+\beta_{3}\;x_{3i}+\beta_{4}x_{4i}+\beta_{5}x_{5i}$$

Where: $Y_{i}$ = ELISA status of a participant (1 = positive, 0 = negative); $X_{1}$ = doses received; $X_{2}$ = season/quarter of enrollment; $X_{3}$ = sex; $X_{4}$ = age in months; $X_{5}$ = disease severity.

VE was estimated using the formula:


VE=(1–OR)*100%.

We adjusted for age, sex, and month of enrollment as potential confounders. Age was classified into <12 months, 12–23 months, 24–59 months, and ≥60 months. Month of enrollment was classified into 4 quarters as January–March, April–June, July–September, and October–December. Further analysis included examination of the duration of protection by measuring effectiveness stratified by age (<12 months and ≥12 months). We assessed the protection against disease of varying severity using a 20-point clinical Vesikari score, classified as less severe (<11) and severe (≥11). To examine Rotarix effectiveness in malnourished children, stunting (low height for age) was used as an indicator of chronic malnutrition, wasting (low weight for height) as an indicator of acute malnutrition, and underweight (low weight for age) as a composite of acute and chronic malnutrition. All were classified as normal (*z* score ≥ −2.0), moderate (*z* score < −2.0 and ≥ −3.0), or severe (*z* score < −3.0) using WHO growth standards [24].

Annual population-level incidence of rotavirus hospitalization was calculated as the number of laboratory-confirmed (ELISA-positive) rotavirus cases identified in each year divided by the corresponding yearly population estimates from the KHDSS area and expressed per 100 000 population [19]. The formula for this was outlined as:


$$\mathrm{Incidence}\_100K\;=\;\left(\frac{Number\;of\;\;rotavirus\;cases}{Population}\right)*\;100,000$$

Incidence estimates were stratified by age group and calendar year.

## RESULTS

### Recruitment and Baseline Characteristics

Between 1 September 2009 and 31 December 2022, a total of 5978 children presented with acute diarrhea illness and were admitted at KCRH. Of these 2743 were admitted pre–vaccine introduction (between 1 September 2009 and 30 June 2014) and 3235 children were admitted post–vaccine introduction (between 1 July 2014 and 31 December 2022) (Figure 1). Among the 2743 children recruited prevaccine, 25.7% (535/2081) tested positive for rotavirus while among 3235 children recruited postvaccine, 13.8% (279/2023) tested positive for rotavirus. For Rotarix VE analysis (1 July 2014 to 31 December 2022), 1104 children (median age, 12 months [interquartile range, 8–18 months]) met our case-control study inclusion criteria. Of the 2131 children who did not meet the inclusion criteria, 742 (35%) were age ineligible, 761 (36%) did not consent to participate, 369 (17%) were missing rotavirus vaccination status information, 236 (11%) had missing ELISA status, and 23 (1%) had no stool sample collected. The flow of participants included in the VE analysis is summarized in Figure 1.

**Figure 1.**
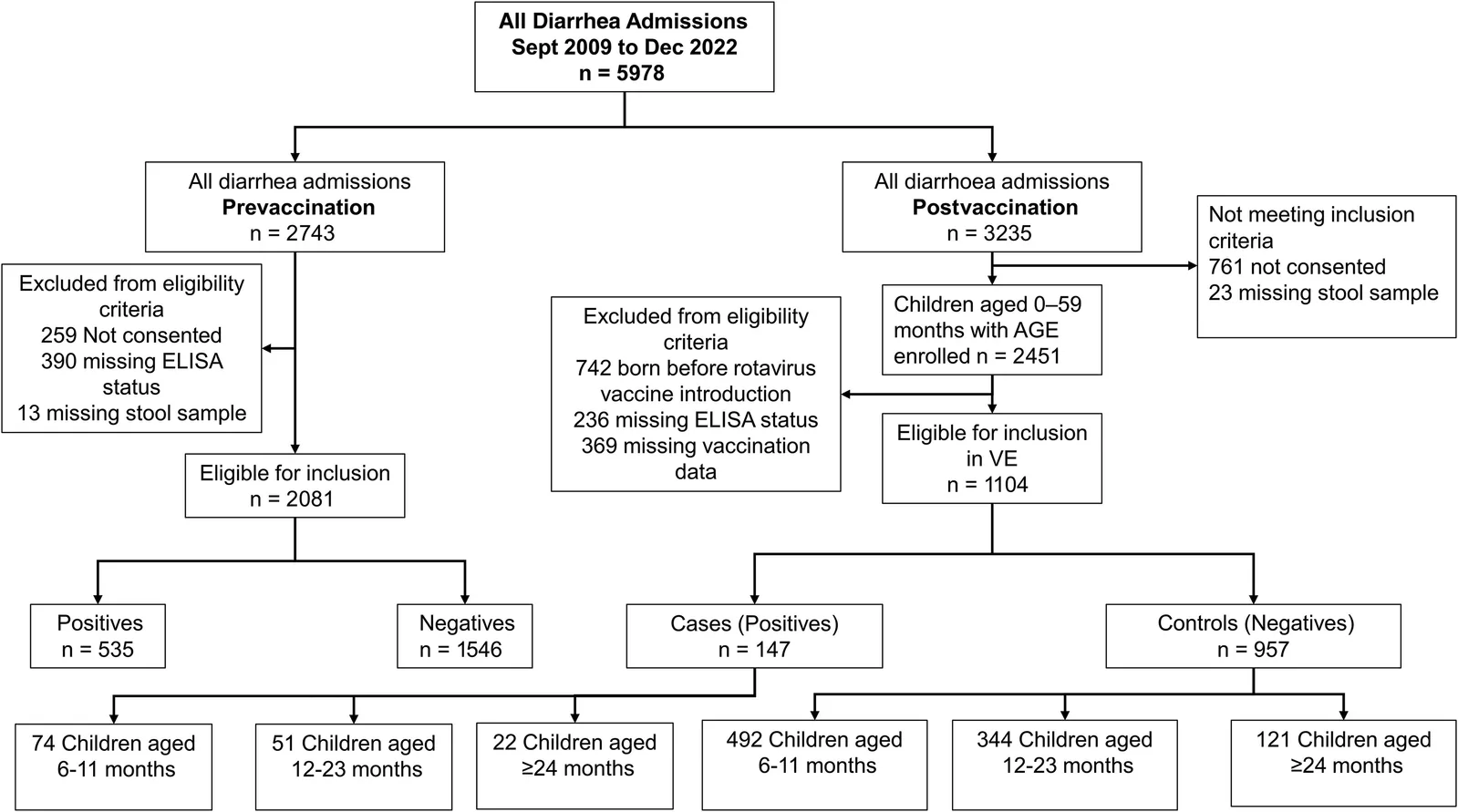
Flowchart of study participants. The breakdown of the number of patients admitted to Kilifi County Referral Hospital, Kenya, with AGE between September 2009 and December 2022. The flow outlines participants eligible for inclusion in the surveillance and the nested test-negative case-control study, detailing the number of rotavirus-positive patients (cases) and rotavirus-negative patients (controls) by study period and by vaccination status. Abbreviations: AGE, acute gastroenteritis; ELISA, enzyme-linked immunosorbent assay; VE, vaccine effectiveness.

### Diarrhea and Rotavirus Seasonal Trends in Kilifi

Figure 2*A* presents monthly temporal trends in diarrheal hospital admissions to KCRH, ELISA-confirmed rotavirus cases over the 13 years of continuous surveillance, and changes in Rotarix coverage. Diarrheal admissions showed seasonal variation with recurrent peaks across years. Similarly, rotavirus cases showed seasonal peaking patterns (frequently between May and November) but at lower peaks. The timing of the rotavirus seasonal peaks was consistent both before and after vaccine introduction, but the magnitude of the peaks declined over time with increasing vaccine uptake. The monthly rotavirus cases ranged from 0 to 29 cases during the prevaccine period and 0 to 31 cases during the Rotarix era. From the annual rotavirus positivity from 2010 to 2022, we observed highest positivity rates prior to vaccine introduction in 2014. Following Rotarix introduction, there was a marked decline in the annual rotavirus positivity, particularly from 2016 onward (except 2018), with the lowest positivity observed in 2020 (coinciding with the coronavirus disease 2019 [COVID-19] pandemic) followed by a rebound in 2021 and 2022 (Figure 2*B*).

**Figure 2.**
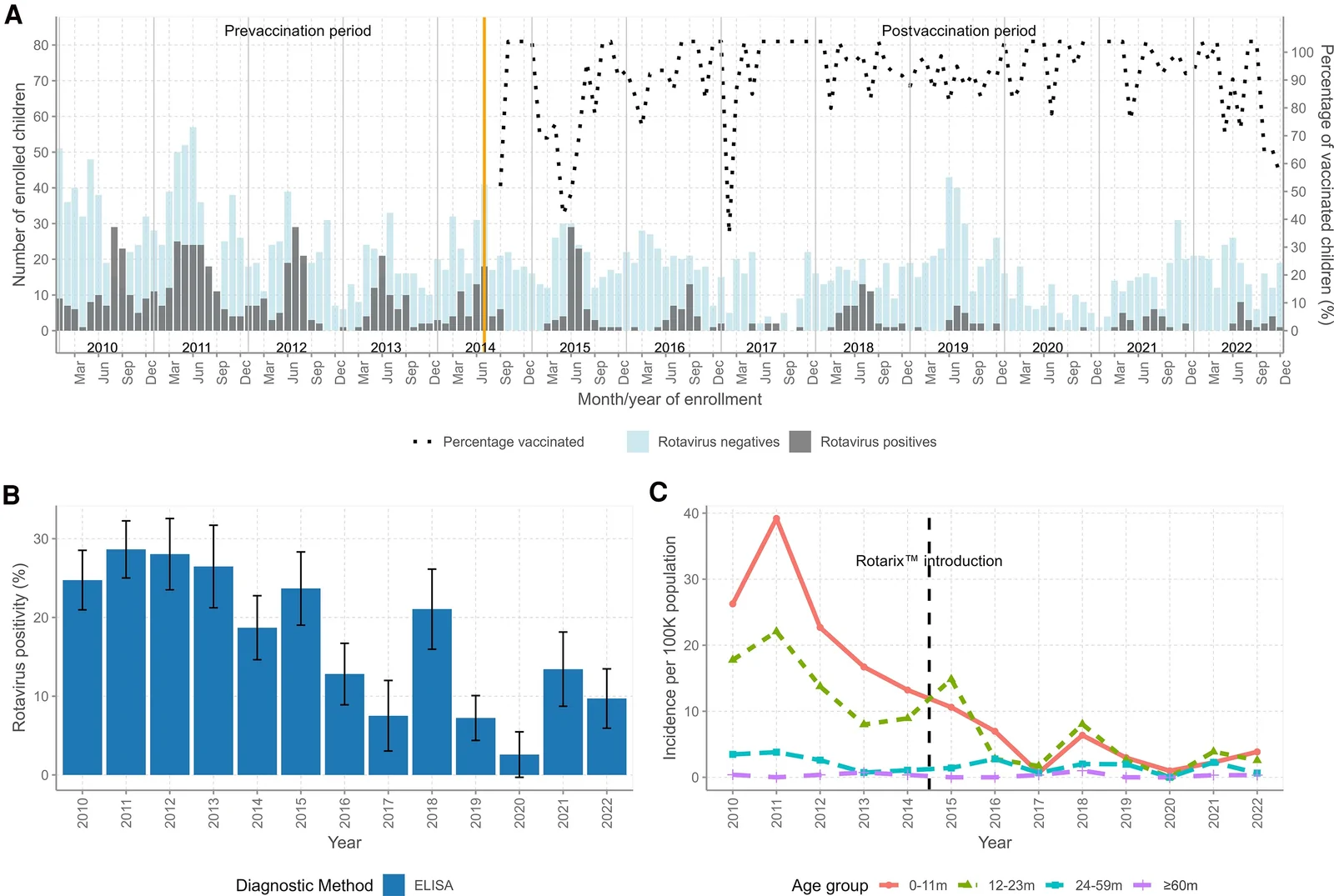
Diarrhea and rotavirus hospitalization trends in Kilifi, Kenya, before and after Rotarix introduction. *A*, Monthly rotavirus cases and vaccine coverage among eligible children (July 2014–December 2022). Bars represent the number of rotavirus-positive (dark gray) and rotavirus-negative (light blue) cases. The dotted line represents Rotarix vaccine coverage among eligible children with known vaccination status. The solid vertical line indicates the introduction of the Rotarix vaccine in July 2014. *B*, Annual rotavirus positivity by enzyme-linked immunosorbent assay (ELISA). *C*, Changes in yearly population-level rotavirus hospitalization incidence (per 100 000 population) stratified by age group within the Kilifi Demographic Surveillance System area.

### Changes in RVA Incidence Over the Years

Figure 2*C* shows annual population-level rotavirus hospitalization incidence per 100 000 population in the KHDSS area stratified by age group from 2009 to 2022. Incidence was highest among infants aged 0–11 months during the prevaccination period, peaking in 2011 with 39 cases per 100 000 per year, compared to 22 cases per 100 000 per year among children aged 11–23 months, while the incidence for the age groups 24–59 months and ≥60 months remained substantially low. Following vaccine introduction, incidence declined sharply across all pediatric age groups, with lowest rates observed in 2017 (note that surveillance in this year was also disrupted by healthcare worker industrial action [25]). Although minor year-to-year fluctuations were observed, particularly among infants, postvaccine incidence remained substantially lower than prevaccine levels. Age-specific changes in rotavirus positivity before and after vaccine introduction are shown in Supplementary Figure 1.

### G and P Genotyping

Eight hundred twenty-two samples tested rotavirus positive between 1 September 2009 and 31 December 2022. G-type and P-type were determined from 746 and 741 samples, respectively (Supplementary Table 2). From these, a total of 8 distinct G genotypes and 4 distinct P genotypes were identified. G1 was predominant in both the prevaccine period (49.3% [266/540]) and postvaccine period (41.3% [85/206]). G3 increased significantly from 2.8% (15/540) in the prevaccine period to 24.3% (50/206) in the postvaccine period. G2 rose from 8.7% (47/540) prevaccine to 22.8% (47/206) in the post–vaccine introduction period. G8 strain had a relatively high prevalence in the prevaccine period of 18.1% (98/540) but was not detected in the postvaccine period. Minor strains (<5%) circulating during the postvaccine period included G10, G12, G29, and G9.

P[8] predominated in both the prevaccine period (65.8% [352/535]) and postvaccine period (68.9% [142/206]). P[4] prevalence was 28.8% (154/535) prevaccine compared to 22.3% (46/206) postvaccine. P[6] circulated in low frequency in the prevaccine period (0.6% [3/535]) and the postvaccine period (1.9% [4/206]). P[14] was detected in the prevaccine period only (0.4% [2/535]).

A total of 24 combinations of G and P genotypes were detected, including mixed genotype infections. The predominant prevaccine genotype G1P[8] (47.1% [252/535]) declined to 28.9% (90/311) postvaccine (Figure 3 and Supplementary Figure 2). A similar decline was observed for G8P[4] (from 16.1% [86/535] to 0% [0/311]). Significant increases were observed in G3P[8] (from 1.1% [6/535] to 31.2% [97/311]) and G2P[4] (from 8.4% [45/535] to 20.3% [63/311]). Other strains circulating post–vaccine introduction were G3P[6] (2.6% [8/311]), G9P[4] (7.4% [23/311]), G10P[4] (0.3% [1/311]), and G2P[8] (0.6% [2/311]) (Supplementary Table 3).

**Figure 3.**
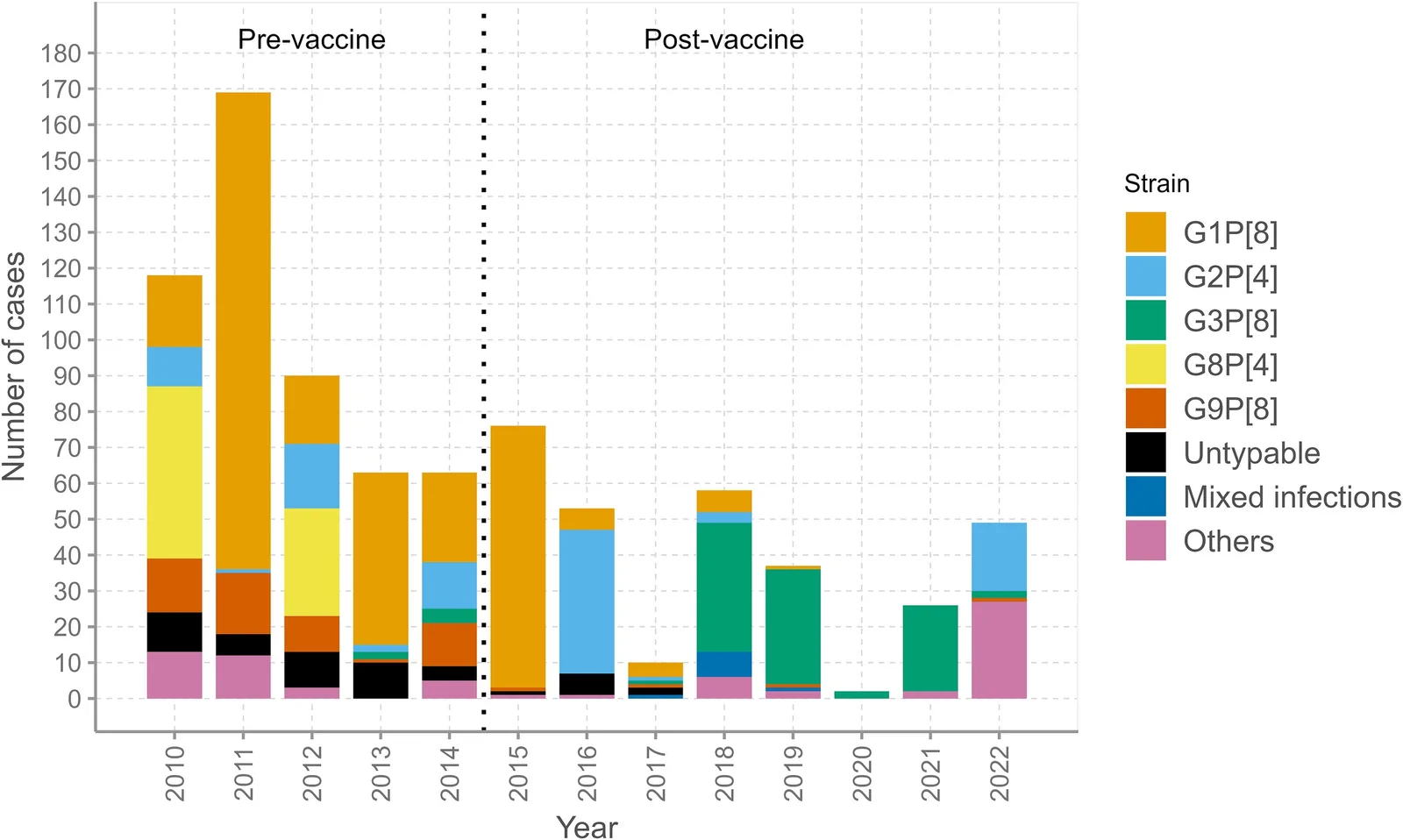
Yearly counts of rotavirus G and P genotype combinations across the pre– and post–Rotarix vaccine introduction periods. The strains included in the “Others” category were G9P[4], G3P[6], G8P[8], G10P[8], G12P[8], G1P[4], G2P[8], G12P[4], G12P[6], G3P[4], G10P[4], G12P[8], and G8P[14].

### Characteristics of Cases and Controls

Among the 1104 test-negative case-control study–eligible children, 147 (13.0%) tested rotavirus positive (cases), while 957 (87.0%) tested rotavirus negative (controls). Among the cases, 120 (82.0%) had received 2 doses, 6 (4.1%) had received 1 dose, and 21 (14.0%) had received no vaccination (zero doses). Among the controls, 843 (88.0%) received 2 doses, 63 (6.7%) received 1 dose, and 51 (5.3%) received zero doses.

G and P genotyping was successfully completed for 144 participants of the 147 VE-eligible cases, including 9 whom had mixed infections. One sample gave an untypeable result (failed P) and 2 had failed both G and P genotyping. The genotyped VE samples fell into G3P[8] (66/147 [44.9%]), G1P[8] (32/147 [21.8%]), G2P[4] (23/147 [15.6%]), G9P[8] (2/147 [1.4%]), G3P[6] (4/147 [2.7%]), G2P[8] (1/147 [0.7%]), and G9P[8] (2/147 [1.4%]). The distribution of the detected genotype by participant vaccination status is provided in Supplementary Table 4.

Between 2014 and December 2021, Rotarix coverage for dose 1 and dose 2 among enrolled children in the study showed consistently high completion rates, with declining coverage in 2022. The proportion of children who received at least 1 dose remained above 80% from 2016 onward, with most children receiving both doses (Supplementary Figure 3). The estimated vaccine coverage in Kenya reported by WHO indicated an increase in coverage from 2014, which was sustained until 2022 when a significant decline occurred (Supplementary Figure 3).

The cases and controls were similar in terms of sex and age, although we observed more males (57% [626/1104]) in the study. Significant differences were observed between cases and controls for the season of enrollment, disease severity category, and the doses received (Table 1). Rotavirus admissions mostly peaked in July–September (82 cases [56%]; χ^2^ test, *P* < .01; Table 1). Among the 5 study-eligible rotavirus-positive children who died, 1 had 0 doses and 4 had received the full 2 doses.

**Table 1.** Characteristics of Rotavirus-Positive (Cases) and Rotavirus-Negative (Controls) Participants by Vaccination Status in the Prevaccine and Postvaccine Periods, Among Children Admitted at Kilifi County Referral Hospital, Kilifi, Kenya (September 2009–December 2022)

Characteristic	Prevaccine Period (September 2009–June 2014)				Postvaccine Period (July 2014–December 2022)			
	RVA^+^ (n = 535)	RVA^–^ (n = 1546)	Total (n = 2081)	*P* Value^a^	RVA^+^ (n = 279)	RVA^–^ (n = 1744)	Total (n = 2023)	*P* Value^a^
Sex				.3				>.9
Female	212 (40)	664 (43)	876 (42)		120 (43)	753 (43)	873 (43)	
Male	323 (60)	882 (57)	1205 (58)		159 (57)	991 (57)	1150 (57)	
Age, mo				<.001				
Median (Q1, Q3)	10 (7, 15)	13 (8, 24)	12 (7, 21)		14 (9, 20)	14 (8, 25)	14 (8, 24)	.2
Age group				<.001				<.001
0–11 mo	321 (60)	721 (47)	1042 (50)		116 (42)	723 (41)	839 (41)	
12–23 mo	176 (33)	439 (28)	615 (30)		121 (43)	557 (32)	678 (34)	
24–59 mo	33 (6.2)	258 (17)	291 (14)		36 (13)	291 (17)	327 (16)	
≥60 mo	5 (0.9)	128 (8.3)	133 (6.4)		6 (2.2)	173 (9.9)	179 (8.8)	
Month of enrollment				<.001				<.001
January–March	87 (16)	374 (24)	461 (22)		9 (3.2)	390 (22)	399 (20)	
April–June	184 (34)	509 (33)	693 (33)		78 (28)	471 (27)	549 (27)	
July–September	197 (37)	269 (17)	466 (22)		161 (58)	442 (25)	603 (30)	
October–December	67 (13)	394 (25)	461 (22)		31 (11)	441 (25)	472 (23)	
Severity category				<.001				<.001
Mild	137 (26)	727 (47)	864 (42)		14 (5.0)	271 (16)	285 (14)	
Moderate	208 (39)	494 (32)	702 (34)		96 (34)	721 (41)	817 (40)	
Severe	190 (36)	325 (21)	515 (25)		169 (61)	752 (43)	921 (46)	
Weight for age				.2				.6
Normal (*z* ≥ −2)	529 (100)	1516 (99)	2045 (99)		278 (100)	1721 (99)	1999 (99)	
Moderately underweight (*z* < −2)	1 (0.2)	11 (0.7)	12 (0.6)		1 (0.4)	12 (0.7)	13 (0.6)	
Severely underweight (*z* < −3)	0 (0)	7 (0.5)	7 (0.3)		0 (0)	11 (0.6)	11 (0.5)	
Weight for height				>.9				<.001
Normal (*z* ≥ −2)	9 (56)	22 (63)	31 (61)		193 (69)	1048 (62)	1241 (63)	
Moderate wasting (*z* < −2)	5 (31)	8 (23)	13 (25)		64 (23)	345 (20)	409 (21)	
Severe wasting (*z* < −3)	2 (13)	5 (14)	7 (14)		21 (7.6)	295 (17)	316 (16)	
Height for age				.2				<.001
Normal (*z* ≥ −2)	13 (81)	21 (57)	34 (64)		214 (77)	1163 (67)	1377 (68)	
Moderate stunting (*z* < −2)	2 (13)	6 (16)	8 (15)		53 (19)	260 (15)	313 (16)	
Severe stunting (*z* < −3)	1 (6.3)	10 (27)	11 (21)		12 (4.3)	314 (18)	326 (16)	
Doses received								<.001
0	…	…	…		26 (9.3)	68 (3.9)	94 (4.6)	
1	…	…	…		7 (2.5)	68 (3.9)	75 (3.7)	
2	…	…	…		126 (45)	862 (49)	988 (49)	
Unknown	…	…	…		120 (43)	746 (43)	866 (43)	

Data are presented as No. (%) unless otherwise indicated.

Abbreviations: RVA^–^, rotavirus group A negative; RVA^+^, rotavirus group A positive; *z*, *z* score.

^a^Pearson χ^2^ test; Wilcoxon rank-sum test; Fisher exact test.

### Rotarix Vaccine Effectiveness

After adjusting for age, sex, month of enrollment, and disease severity, the overall effectiveness among age-eligible children was 68% (95% CI, 42%–82%) for 2 doses and 76% (95% CI, 35%–92%) for 1 dose (Table 2). For children aged <12 months, 2-dose effectiveness was 83% (95% CI, 62%–93%) and 1-dose effectiveness was 79% (95% CI, 28%–95%). For children aged ≥12 months, 2-dose effectiveness was 38% (95% CI, −106% to 78%) and 1-dose VE was 65% (95% CI, −102% to 96%). A formal likelihood ratio test found no interaction between vaccination status and age group (*P* = .239). Sensitivity analyses using a matched case-control analysis and narrower age cut-offs are presented in Supplementary Tables 6 and 7.

**Table 2.** Crude and Adjusted Rotarix Vaccine Effectiveness Estimates Among Children Admitted to Kilifi County Referral Hospital With Rotavirus Gastroenteritis (July 2014–December 2022)

Age Group and Doses	Cases (n = 147)	Controls (n = 957)	Crude VE (95% CI)	Adjusted VE^a^ (95% CI)
All age eligible				
95 0 doses (Reference)	21	51	Reference	Reference
1 dose	6	64	77% (42%–92%)	76% (35%–92%)
2 doses	120	842	65% (39%–80%)	68% (42%–82%)
Age <12 mo				
0 doses (Reference)	16	28	Reference	Reference
1 dose	4	42	83% (49%–96%)	79% (28%–95%)
2 doses	54	421	76% (55%–88%)	83% (62%–93%)
Age ≥12 mo				
0 doses (Reference)	5	23	Reference	Reference
1 dose	2	22	58% (−117% to 94%)	65% (−102% to 96%)
2 doses	66	421	28% (−121% to 71%)	38% (−106% to 78%)
Less severe (Vesikari score <11)				
0 doses	11	22	Reference	Reference
2 doses	38	438	83% (64%–92%)	85% (65%–93%)
Severe (Vesikari score ≥11)				
0 doses	10	29	Reference	Reference
2 doses	82	404	41% (−31% to 72%)	47% (−22% to 76%)

Abbreviations: CI, confidence interval; VE, vaccine effectiveness.

^a^Adjusted for season (month of enrollment), age in months, sex, and disease severity (except where stratified by severity).

The 2-dose VE against less severe gastroenteritis (Vesikari score <11) was 85% (95% CI, 65%–93%) compared to 47% (95% CI, −22% to 76%) against severe gastroenteritis (Vesikari score ≥11). For normal weight for age, 2-dose effectiveness was 66% (95% CI, 38%–81%). For children with a normal height for age, 2-dose VE was 56% (95% CI, 10%–78%) compared to 87% (95% CI, 54%–97%) of those with moderate stunting and 68% (95% CI, −659% to 97%) among those with severe stunting. Effectiveness was 78% in children with normal weight for height (95% CI, 53%–89%), 33% among children with moderate wasting (95% CI, −243% to 83%), and −1% among children with severe wasting (95% CI, −1917% to 73%) (Supplementary Table 5).

## DISCUSSION

Over 8.5 years of routine Rotarix vaccination, we estimated its 2-dose VE to be 68% (95% CI, 42%–82%) against hospitalized rotavirus gastroenteritis. This finding is comparable with initial estimates 2.5 years after Rotarix introduction into the NIP of Kenya (64% [95% CI, 35%–80%]) [4]. Following Rotarix vaccine introduction in Kenya in July 2014, the proportion of rotavirus hospitalized cases among our study population declined by 46%, decreasing from 25.7% pre–vaccine introduction to 13.8% post–vaccine introduction. While the highest reduction in incidence occurred among infants aged 0–11 months, children aged 12–23 months also experienced lower incidence rates even though VE estimates were comparatively lower in this age group. Percent positivity also decreased across the postvaccine period, indicating an overall reduction in rotavirus disease burden.

In an early evaluation by Khagayi et al [4], similar VE was demonstrated among children aged <12 months and ≥12 months (67% vs 72%). However, our long-term analysis found higher point estimates of VE among the <12 months compared to ≥12 months (83% vs 38%). This apparent reduction in protection for children ≥12 months aligns with observations in other medium- and high-mortality countries where effectiveness tends to wane after the first year of life [26, 27]. Findings from Jonesteller et al [28] showed a median Rotarix effectiveness of 84%, 75%, and 57% in countries with low, medium, and high child mortality, respectively, and that effectiveness tended to decline in the second year of life. Rotavirus vaccine immunity waning is an area of current active research including potential correction measures, for example, by administering a third dose or through alternative routes.

Furthermore as highlighted by Pitzer et al [29, 30], an important limitation of the test-negative case-control study design when estimating VE during the second year of life is the influence of naturally acquired immunity. Unvaccinated children are highly likely to experience natural, including asymptomatic, rotavirus infection during infancy, which confers partial protection against severe disease later. This reduces the immunologic differences between vaccinated and unvaccinated children over time, resulting in lower observed VE estimates in older age groups even without true waning of vaccine-derived immunity. Additionally, VE estimates in older age groups may be affected by baseline heterogeneity in susceptibility and disease severity across populations, which, as Lewnard et al [31] demonstrated, can contribute to lower observed vaccine protection in high-incidence settings.

Congruent with findings from similar studies and in the earlier analysis [4], we observed higher VE estimates among children with less severe gastroenteritis (85%) compared with those with severe gastroenteritis (Vesikari score ≥11) (47%). This apparent lower effectiveness against severe disease is counterintuitive as rotavirus vaccines are designed primarily to prevent severe outcomes. However, this finding must be interpreted cautiously as it is limited by a relatively small number of severe cases in our cohort and the resulting statistical uncertainty (95% CI, −22% to 76%). Similarly, while effectiveness was maintained in children with normal nutritional status and those with moderate stunting, our estimates among underweight and wasted children showed limited VE that was highly uncertain due to small sample sizes. Nonetheless, our overall sustained Rotarix effectiveness aligns with long-term reports from other African countries including Madagascar (61%), Malawi (62%), and Tanzania (53%) [32–34], as well as sustained hospital incidence declines in Zambia [35] and Ghana [36].

We observed significant genetic diversity in strains circulating post–Rotarix introduction. Pre–vaccine introduction, the most frequent RVA genotypes circulating in Kenya were G1P[8], G8P[4], G9P[8], G2P[4], and G3P[6] (3.1%) [21]. Post–vaccine introduction, we noted prominent detection of heterologous strains G3P[8] and G2P[4] alongside the homotypic G1P[8]. This findings reflect findings from Seheri et al from 2010–2015 across 15 eastern and southern African countries where G1P[8] was leading (23.8%), followed by G2P[4] (11.8%), G9P[8] (10.4%), G12P[8] (4.9%), G2P[6] (4.2%), and G3P[6] (3.7%) [37]. Similar strain variations were observed in Madagascar [38], Ghana [39], Zambia, and Zimbabwe [37–41]. Our findings regarding changes in strain predominance, particularly during the COVID-19 pandemic and postpandemic periods, should be interpreted with caution due to the small number of cases available for genotyping during those years.

Our study has limitations. First, several potential participants in the study were excluded due to absence of parent/guardian consent to participate, missing vaccination information, and unknown rotavirus status. Second, during the 13-year surveillance period, multiple industrial actions by healthcare workers (mainly nurses and doctors) happened, which impacted health-seeking behavior, recruitment, and sample collection. Third, the COVID-19 pandemic significantly disrupted hospital services and healthcare-seeking behaviors (eg, vaccination services). Finally, because we relied on routinely collected surveillance data, were unable to adjust our effectiveness estimates for a number of potential unmeasured confounders such as breastfeeding status, socioeconomic status, coinfections, or difference in healthcare-seeking behaviors.

## CONCLUSIONS

The Rotarix vaccine maintained its modest effectiveness in protecting children against rotavirus severe AGE during its 8.5 years of routine use in coastal Kenya. While overall disease incidence fell sharply, the apparent decline in VE in children aged >1 year reinforces the need for tailored strategies to sustain protection against rotavirus disease beyond the first year of life. Furthermore, the pronounced detection of heterologous strains such as G3P[8] and G2P[4], albeit with our limited sample size, highlights the need for ongoing genomic surveillance to monitor the VE of rotavirus vaccines. This is especially reinforced Kenya's recent programmatic switch to the 3-dose monovalent Rotavac vaccine, the effectiveness of which must be closely monitored as it becomes more widely utilized across the African continent.

## Supplementary Material

ofag674_Supplementary_Data

## Supplementary Data

Supplementary materials are available at *Open Forum Infectious Diseases* online. Consisting of data provided by the authors to benefit the reader, the posted materials are not copyedited and are the sole responsibility of the authors, so questions or comments should be addressed to the corresponding author.
