# Supplementary material for: Long-term Rotarix Vaccine Effectiveness in Coastal Kenya, Estimated From Child Hospital Admissions Data From July 2014 to December 2022
Source: Open Forum Infect Dis. 2026 Oct 10;13(10):ofag674. doi: 10.1093/ofid/ofag674 (PMC13684409; doi:10.1093/ofid/ofag674)
Supplement: Supplementary file 1 [file ofag674_supplementary_data.docx]

**Supplementary Material**

**Article title**

Long-term Rotarix™ vaccine effectiveness in coastal Kenya, estimated from child hospital admissions data from July 2014 to December 2022

**Authors**

Zadock Kipkirui, Charity Wamwea, Esther N. Katama, Robinson Cheruiyot, Martin Mutunga, Agustus Kea, Arnold W. Lambisia, D. James Nokes, Umesh D. Parashar, Jacqueline E. Tate, Eunice W. Kagucia & Charles N. Agoti

**Supplementary Tables**

**Supplementary Table 1.** Estimation of required sample sizes for the test-negative case-control study design different vaccine coverage

| Vaccine Coverage | Cases | Controls | Total |
| --- | --- | --- | --- |
| 60% | 100 | 199 | 299 |
| 70% | 107 | 213 | 320 |
| 80% | 132 | 263 | 395 |
| 90% | 219 | 437 | 656 |
| 95% | 401 | 801 | 1,202 |

**Supplementary Table 2.** Distribution of individual G and P genotypes among rotavirus positive successfully typed cases by study period.

|  | Pre-vaccine period  Sep 2009- Jun 2014 | Post vaccine period  July 2014- Dec 2022 | Total |
| --- | --- | --- | --- |
| G-type | **n = 540** | **n = 206** | **N = 746** |
| G1 | 266 (49.3%) | 85 (41.3%) | 351 (47.1%) |
| G8 | 98 (18.1%) | - | 98 (13.1%) |
| G2 | 47 (8.7%) | 47 (22.8%) | 94 (12.6%) |
| G9 | 61 (11.3%) | 6 (2.9%) | 67 (9.0%) |
| G3 | 15 (2.8%) | 50 (24.3%) | 65 (8.7%) |
| G12 | 14 (2.6%) | 3 (1.5%) | 17 (2.3%) |
| G10 | 6 (1.1%) | - | 6 (0.8%) |
| G29 | 1 (0.2%) | - | 1 (0.1%) |
| G1/3 | - | 3 (1.5%) | 3 (0.4%) |
| G1/3/9 | - | 1 (0.5%) | 1 (0.1%) |
| G3/12 | - | 2 (1.0%) | 2 (0.3%) |
| G3/9 | - | 1 (0.5%) | 1 (0.1%) |
| Gx (Untypeable) | 32 (5.9%) | 8 (3.9%) | 40 (5.4%) |
|  |  |  |  |
| P-type | **n = 535** | **n = 206** | **N = 741** |
| P[8] | 352 (65.8%) | 142 (68.9%) | 494 (66.6%) |
| P[4] | 154 (28.8%) | 46 (22.3%) | 200 (27.0%) |
| P[6] | 3 (0.6%) | 4 (1.9%) | 7 (0.9%) |
| P[14] | 2 (0.4%) | - | 2 (0.3%) |
| P[4][8] | - | 1 (0.5%) | 1 (0.1%) |
| P[6][8] | - | 6 (2.9%) | 6 (0.8%) |
| P[x] (Untypeable) | 24 (4.5%) | 7 (3.4%) | 31 (4.1%) |

Footnote: Untypeable G-type accounted by Gx 40(5.5%), while P-type by P[x] = 31 (4.3%)

**Supplementary Table 3.** Distribution of rotavirus G and P genotypes before and after vaccine introduction in Coastal, Kenya, 2009-2022

| Strain | Pre-vaccine | Post-vaccine | Total |
| --- | --- | --- | --- |
| G1P[8] | 252 (47.1%) | 90 (28.9%) | 342 (40.4%) |
| G2P[4] | 45 (8.4%) | 63 (20.3%) | 108 (12.8%) |
| G3P[8] | 6 (1.1%) | 97 (31.2%) | 103 (12.2%) |
| G8P[4] | 86 (16.1%) | - | 86 (10.2%) |
| G9P[8] | 56 (10.5%) | 4 (1.3%) | 60 (7.1%) |
| G9P[4] | 4 (0.7%) | 23 (7.4%) | 27 (3.2%) |
| G3P[6] | 1 (0.2%) | 8 (2.6%) | 9 (1.1%) |
| G12P[8] | 6 (1.1%) | 1 (0.3%) | 7 (0.8%) |
| G8P[8] | 5 (0.9%) | 2 (0.6%) | 7 (0.8%) |
| G10P[8] | 6 (1.1%) | - | 6 (0.7%) |
| G1P[4] | 5 (0.9%) | - | 5 (0.6%) |
| G12P[6] | 2 (0.4%) | 2 (0.6%) | 4 (0.5%) |
| G2P[8] | 1 (0.2%) | 2 (0.6%) | 3 (0.4%) |
| G3P[4] | 2 (0.4%) | - | 2 (0.2%) |
| G1/3P[8] | - | 2 (0.6%) | 2 (0.2%) |
| G12P[4] | 2 (0.4%) | - | 2 (0.2%) |
| G10P[4] | - | 1 (0.3%) | 1 (0.1%) |
| G8P[14] | 1 (0.2%) | - | 1 (0.1%) |
| G3/12P[6][8] | - | 2 (0.6%) | 2 (0.2%) |
| G1/3/9P[6][8] | - | 1 (0.3%) | 1 (0.1%) |
| G1/3P[6][8] | - | 1 (0.3%) | 1 (0.1%) |
| G3/9P[6][8] | - | 1 (0.3%) | 1 (0.1%) |
| G3P[6][8] | - | 1 (0.3%) | 1 (0.1%) |
| G9P[4][8] | - | 1 (0.3%) | 1 (0.1%) |
| Untypable | 55 (10.3%) | 9 (2.9%) | 64 (7.6%) |
| Total | 535 (100.0%) | 311 (100.0%) | 846 (100.0%) |

Footnote: Untypeable comprised of G1P[x] (n = 8), G2P[x] (n = 1), G3P[x] (n = 6), G8P[x] (n = 4), G9P[x] (n = 3), G12P[x] (n = 4), GxP[4] (n = 11), G29P[x] (n = 1) and GxP[8] (n = 26).

**Supplementary Table 4.** Distribution of genotypes among vaccinated and unvaccinated rotavirus cases included in the vaccine effectiveness study (July 2014 – December 2022)

| Strain | 0 Doses  (n = 21) | 1 Dose  (n = 6) | 2 Doses  (n = 120) | Total  (n = 147) |
| --- | --- | --- | --- | --- |
| **G3P[8]** | 3 (14.3%) | 3 (50.0%) | 60 (50.0%) | 66 (44.9%) |
| **G2P[4]** | 3 (14.3%) | - | 20 (16.7%) | 23 (15.6%) |
| **G1P[8]** | 9 (42.9%) | 3 (50.0%) | 20 (16.7%) | 32 (21.8%) |
| **G9P[4]** | 2 (9.5%) | - | 3 (2.5%) | 5 (3.4%) |
| **G3P[6]** | 2 (9.5%) | - | 2 (1.7%) | 4 (2.7%) |
| **G12P[6]** | 1 (4.8%) | - | 1 (0.8%) | 2 (1.4%) |
| **G9P[8]** | - | - | 2 (1.7%) | 2 (1.4%) |
| **G1/3P[8]** | - | - | 2 (1.7%) | 2 (1.4%) |
| **G3/12P[6][8]** | - | - | 2 (1.7%) | 2 (1.4%) |
| **G2P[8]** | - | - | 1 (0.8%) | 1 (0.7%) |
| **G3/9P[6][8]** | - | - | 1 (0.8%) | 1 (0.7%) |
| **G1/3/9P[6][8]** | - | - | 1 (0.8%) | 1 (0.7%) |
| **G1/3P[6][8]** | - | - | 1 (0.8%) | 1 (0.7%) |
| **G3P[6][8]** | 1 (4.8%) | - | - | 1 (0.7%) |
| **G9P[4][8]** | - | - | 1 (0.8%) | 1 (0.7%) |
| **G3P[x] (untypeable)** | - | - | 1 (0.8%) | 1 (0.7%) |
| **Missing genotyping** | - | - | 2 (1.7%) | 2 (1.4%) |
| **Total** | 21 (100.0%) | 6 (100.0%) | 120 (100.0%) | 147 (100.0%) |

Footnote: Untypeable comprised of G3P[x]

**Supplementary Table 5.** Crude and Adjusted Rotarix™ vaccine effectiveness estimates stratified by nutritional status for the period July 2014 to December 2022.

|  | **Cases** | **Controls** | **Crude vaccine effectiveness (95% CI)** | **Adjusted vaccine effectiveness (95% CI)** |
| --- | --- | --- | --- | --- |
| **Normal weight for age**  **(z ≥ -2)** |  |  |  |  |
| 0 doses | 20 | 50 | Reference |  |
| 2 doses | 120 | 837 | 65% (38% to 80%) | 66% (95% CI, 38% to 81%) |
| **Normal height for age** |  |  |  |  |
| 0 doses | 13 | 36 | Reference |  |
| 2 doses | 93 | 597 | 58% (15% to 79%) | 56% (10% to 78%) |
| **Moderate stunting** |  |  |  |  |
| 0 doses | 7 | 7 | Reference |  |
| 2 doses | 21 | 128 | 84% (50% to 95%) | 87% (54% to 97%) |
| **Severe stunting** |  |  |  |  |
| 0 doses | 1 | 8 | Reference |  |
| 2 doses | 6 | 113 | 60% (-703% to 94%) | 68% (-659% to 97%) |
| **Moderate/ Severe stunting** |  |  |  |  |
| 0 doses | 8 | 15 | Reference |  |
| 2 doses | 27 | 241 | 79% (58% to 89%) | 85% (54% to 95%) |
| **Normal weight for height** |  |  |  |  |
| 0 doses | 16 | 25 | Reference |  |
| 2 doses | 74 | 547 | 79% (58% to 89%) | 79% (55% to 90%) |
| **Moderate wasting** |  |  |  |  |
| 0 doses | 3 | 14 | Reference |  |
| **2 doses** | 33 | 172 | 16% (-277% to 74%) | 33% (-243% to 83%) |
| **Severe wasting** |  |  |  |  |
| **0 doses** | 1 | 12 | reference |  |
| 2 doses | 13 | 116 | -22% (- 2226% to 79%) | -1% (-1917% to 85%) |

**Supplementary table 6**. Matched estimates of Vaccine effectiveness among all age eligible, children aged <12 months and children aged ≥12 months.

|  | Unmatched analysis (adjusted for seasonality) | | | | Matched case-control analysis | | | |
| --- | --- | --- | --- | --- | --- | --- | --- | --- |
|  | Cases | Controls | Crude VE (95% CI) | Adjusted VE (95% CI) | Cases | Controls | Crude VE (95% CI) | Adjusted VE (95% CI) |
| All age eligible |  |  |  |  |  |  |  |  |
| 0 doses (Reference) | 21 | 51 | Reference | Reference | 20 | 13 | Reference | Reference |
| 1 dose | 6 | 64 | 77% (42%, 92%) | 76% (35%, 92%) | 6 | 15 | 72% (12%, 91%) | 73(14%,91%) |
| 2 doses | 120 | 842 | 65% (39%, 80%) | 68% (42%, 82%) | 116 | 212 | 63% (18%, 84%) | 65% (20%, 84%) |
| Age <12 months |  |  |  |  |  |  |  |  |
| 0 doses (Reference) | 16 | 28 | Reference | Reference | 7 | 14 | Reference | Reference |
| 1 dose | 4 | 42 | 83% (49%, 96%) | 79% (28%, 95%) | 4 | 7 | 96 (51%,100%) | 96% (48%,100%) |
| 2 doses | 54 | 421 | 76% (55%, 88%) | 83% (62%, 93%) | 48 | 86 | 92% (39%, 99%) | 94% (41%, 99%) |
| Age ≥ 12 months |  |  |  |  |  |  |  |  |
| 0 doses (Reference) | 5 | 23 | Reference | Reference | 5 | 2 | Reference | Reference |
| 1 dose | 2 | 22 | 58% (-117%, 94%) | 65 (-102%, 96%) | 2 | 2 | 83% (-222%, 99%) | 72% (-514%, 99%) |
| 2 doses | 66 | 421 | 28% (-121%, 71%) | 38% (-106%,78%) | 63 | 111 | 86 (-26%, 98%) | 76% (-138%, 98%) |

VE, Vaccine Effectiveness; CI, confidence interval. Unmatched VE estimates are adjusted for calendar year, month of enrolment, age in months, sex and disease severity; Matched VE estimates are derived using conditional logistic regression, matching cases and controls 1:2 on age category and enrolment season.

**Supplementary table 7.** Adjusted Rotarix™ vaccine effectiveness estimates among different age-cutoffs for children admitted to KCRH with rotavirus gastroenteritis (July 2014 to December 2022).

| Cohort and vaccination status | Cases (n) | Controls (n) | Crude VE (95% CI) | Adjusted VE (95% CI) |
| --- | --- | --- | --- | --- |
| All age eligible | | | | |
| 0 dose (Reference) | 21 | 51 | Reference | Reference |
| 1 dose | 6 | 64 | 77% (42%,92%) | 76% (35%,92%) |
| 2 doses | 120 | 842 | 65% (39%,80%) | 68% (42%,82%) |
| Any dose | 147 | 957 | 66% (41%,80%) | 69% (43%,83%) |
| Children aged <12 months | | | | |
| 0 dose (reference) | 16 | 28 | Reference | Reference |
| 1 dose | 4 | 42 | 83% (49%,96%) | 79% (28%,95%) |
| 2 doses | 54 | 421 | 76% (55%,88%) | 83% (62%,93%) |
| Children aged ≥ 12 months | | | | |
| 0 dose (reference) | 5 | 23 | Reference | Reference |
| 1 dose | 2 | 22 | 58% (-117%, 94%) | 65% (-102%,96%) |
| 2 doses | 66 | 421 | 28% (-121%, 71%) | 38% (-106%,78%) |
| Children aged 12-24 months | | | | |
| 0 dose (reference) | 2 | 16 | Reference | Reference |
| 1 dose | 1 | 17 | 53% (-438%, 98%) | 68% (-320%, 99%) |
| 2 doses | 48 | 311 | -23% (-696%, 66%) | -1% (-597%, 76%) |
| Children aged 24-36 months | | | | |
| 0 doses reference | 0 | 3 | Reference | Reference |
| 1 dose | 0 | 4 | - | - |
| 2 doses | 11 | 66 | - | - |
| Children aged 36-60 months | | | | |
| 0 doses reference | 2 | 3 | Reference | Reference |
| 1 dose | 1 | 2 | 25% (-1410%, 98%) | 60% (-3305%, 100%) |
| 2 doses | 6 | 33 | 73% (-139%, 96%) | 90% (-91%, 100%) |
| Children aged >60 months | | | | |
| 0 doses | 1 | 1 | Reference | Reference |
| 1 dose | - | - | - | - |
| 2 doses | 1 | 9 | - | - |
| Children aged 6-59 months | | | | |
| 0 dose (reference) | 15 | 38 | Reference | Reference |
| 1 dose | 5 | 42 | 70% (14%,91%) | 68% (25%,91%) |
| 2 doses | 110 | 707 | 61 (24%,79%) | 62% (22%,80%) |
| Children aged 6-11 months | | | | |
| 0 dose (reference) | 11 | 14 | Reference | Reference |
| 1 dose | 3 | 19 | 80% (22%,96%) | 79% (4%,96%) |
| 2 doses | 42 | 251 | 79% (49%,91%) | 83% (51%,94%) |
| Children aged 12-17 months | | | | |
| 0 dose (reference) | 1 | 9 | Reference | Reference |
| 1 dose | 1 | 8 | -13% (-3071%,96%) | 20% (-2508%,96%) |
| 2 doses | 25 | 178 | -26% (-2280%,78%) | -25% (-2447%,82%) |
| Children aged 18-23 months | | | | |
| 0 dose (reference) | 1 | 5 | Reference | Reference |
| 1 dose | 0 | 8 | - | - |
| 2 doses | 18 | 99 | 9% (-1696%,86%) | -28% (-2780%,86%) |
| Children aged 12-23 months | | | | |
| 0 dose (reference) | 2 | 15 | Reference | Reference |
| 1 dose | 1 | 16 | 53% (-439%,98%) | 67% (-321%,99%) |
| 2 doses | 46 | 304 | -13% (-635%,69%) | -3% (-613%, 78%) |
| Children aged 24 months and above | | | | |
| 0 dose (reference) | 3 | 7 | Reference | Reference |
| 1 dose | 1 | 6 | 61% (-303%,98%) | 65% (-390%,99%) |
| 2 doses | 18 | 108 | 61% (-93%, 90%) | 72% (-72%,95%) |

**Supplementary Figures**

**Supplementary Figure 1**. Age-specific rotavirus A (RVA) cases over time in Coastal Kenya (2010–2022). Temporal trends in RVA cases stratified by age group, highlighting changes in rotavirus burden pre-vaccination and post-vaccination.

**Supplementary Figure 2.** Yearly distribution of rotavirus GP genotype combination strains across the pre- and post-Rotarix™ vaccine introduction years. The strains included in the "Others" category comprise minor circulating genotypes comprise minor circulating genotypes: G9P[4], G3P[6], G8P[8], G10P[8], G12P[8], G1P[4],G2P[8], G12P[4], G12P[6], G3P[4], G10P[4], G12P[8] and G8P[14].

**Supplementary Figure 3**. Rotavirus vaccine coverage, 2014-2022 in Coastal Kenya. Panel A shows the annual proportion of age-eligible children receiving 0 doses orange solid line, 1 dose green dotted line, and 2 doses blue dashed line of the Rotarix™ vaccine among children with known vaccination status. Panel B, overall annual vaccine coverage (%) black solid line.
